# Supplementary material for: Dopamine and cortical neurons with different Parkinsonian mutations show variation in lysosomal and mitochondrial dysfunction
Source: NPJ Parkinsons Dis. 2025 Jun 20;11:177. doi: 10.1038/s41531-025-01048-2 (PMC12181412; doi:10.1038/s41531-025-01048-2)
Supplement: Supplementary file 1 — Supplementary data revised [file 41531_2025_1048_MOESM1_ESM.pdf]

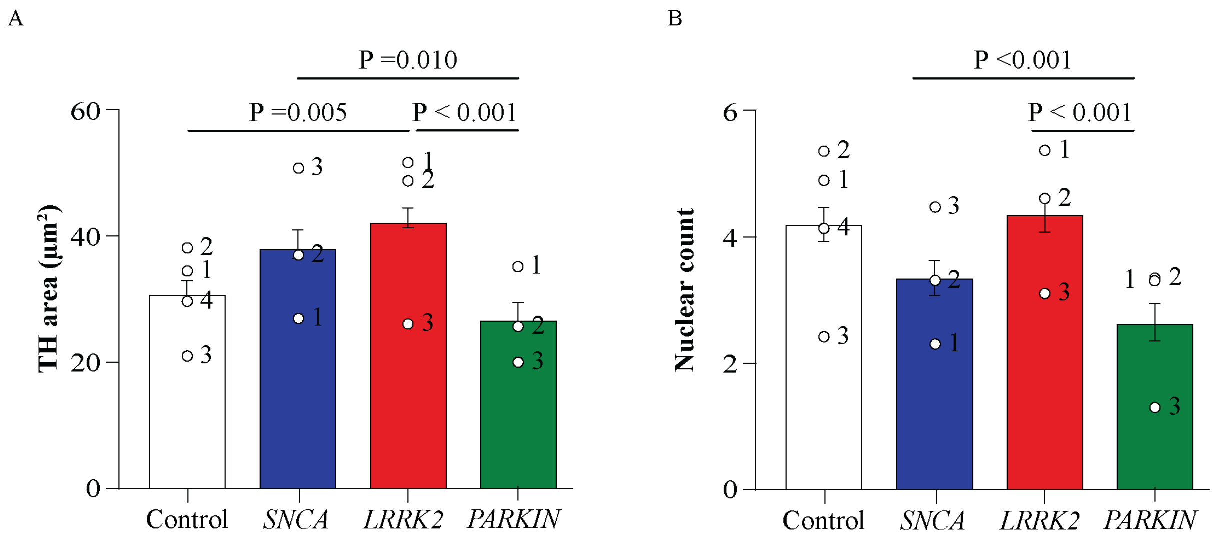

Supplementary Figure 1. A) Average TH area (µm2) calculated for each cell line of the different mutation groups in VMDA neurons. **B**) Nuclei count calculated for each cell line of the different mutation groups in VMDA neurons. Graphs show mean ± SEM. Each datapoint represents the mean value for each cell line derived from n=3 biological replications (each performed in at least duplicate).

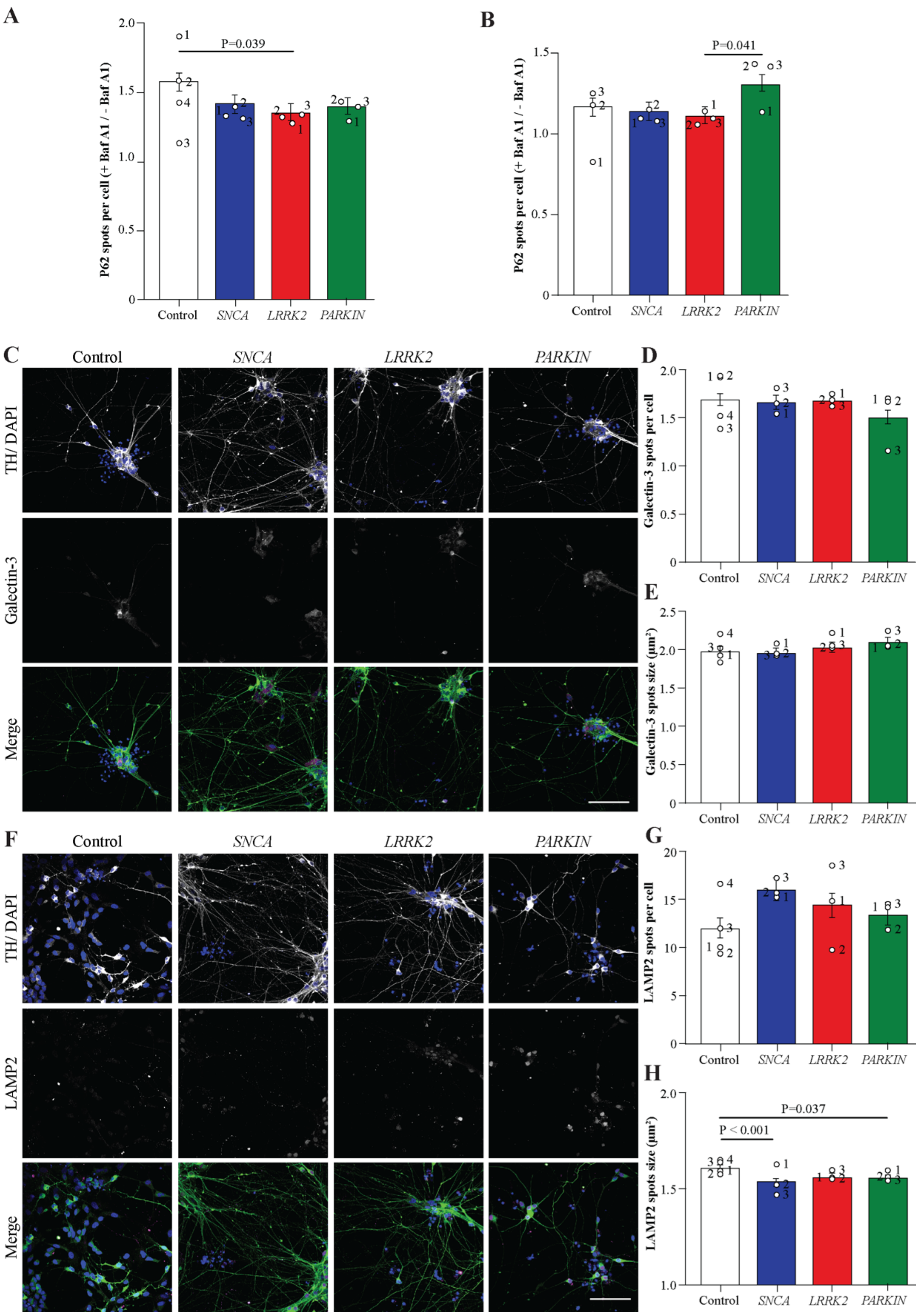

Supplementary Figure 2. A) The ratio of P62 spots in Bafilomycin A1 treated cells / non-treated for each cell line of the different mutation groups in VMDA neurons. **B**) The ratio of P62 spots in Bafilomycin A1 treated cells / non-treated for each cell line of the different mutation groups in cortical neurons. **C**) Representative images of VMDA neurons immunostained with Galectin-3 (magenta), MAP2 (green) and DAPI (blue). The number (**D**) and size (**E**) of Galectin-3 spots in the different mutation groups of VMDA neurons. **F**)

Representative images of VMDA neurons immunostained with LAMP2A (magenta), TH (green) and DAPI (blue). The number (**G**) and size (**H**) of LAMP2A spots in the different mutation groups of VMDA neurons The scale bar in all images is 200 µm. Graphs show mean ± SEM. Each datapoint represents the mean value for each cell line derived from n=3 biological replications (each performed in at least duplicate).

Group	Cell line	RRID	Identifier	Age/sex	Disease	Mutation
**Control**	RM3.5	NA	Control-1	NA	-	-
	PPMI 11450-107	CVCL_D5Q0	Control-2	64/M	-	-
	PPMI 11302-101	CVCL_D5NM	Control-5	76/M	-	-
	KOLF2-1J	CVCL_B5P3	Control-3	55-59/M	-	-
**LRRK2**	PPMI 11576-101	CVCL_D5SB	LRRK2-1	65/M	Unaffected	R1441G
	PPMI 14557-107	CVCL_D5S9	LRRK2-2	42/F	PD	R1441G
	PPMI 14555-107	CVCL_D5RY	LRRK2-3	67/M	PD	R1441G
**PARKIN**	01-060 C9	CVCL_E3WU	PARKIN-1	58/F	PD	Ex3 del +int SNV
	09-090 C18	CVCL_D6I6	PARKIN-2	39/F	PD	Ex2 del +ex2-5 del
	PK6 C2 / C4	CVCL_C8H8	PARKIN-3	49/M	PD	R275W + 132kb del
**SNCA**	PPMI 11555-104	CVCL_D5SL	SNCA-1	36/M	PD	A53T
	PPMI 11557-103	CVCL_D5S0	SNCA-2	53/M	PD	A53T
	PPMI 11556-110	CVCL_D5RU	SNCA-3	54/F	PD	A53T

Table S1. Cell line details. Demographic and mutation information for cell lines used in this study. NA = not available.

PRKN 09-090 C18

Minimum iPSC Quality Control Panel			
**Genomic Baseline QC**	**Pass QC?**	**Method used**	**Results**
Genotyping	Yes	WGS	Exon deletion breakpoints confirmed
Karyotype	Yes	High-denity SNP array (Illumina GSA24)	Normal (part of the Lab resource manuscript submission to Stem Cell Research)
Pluripotent phenotype	Yes	PSC marker staining	iPSC colony morphology,nuclear OCT4/POU5F1, NANOG, manuscript submitted (Lab resource- Stem Cell Re
Cell Line Identity	Yes	STR (10 markers)	Cell line genetic identity with source fibroblasts confirmed
Mycoplasma Testing	Yes	MycoAlert ELISA	Data not shown
Clonality	Yes	double-picked, +10 passage screened	Data not shown
Stability	Yes	30+ passage screened	Data not shown
**Extended iPSC Quality Control Panel**			
**Genomic Baseline QC**	**Pass QC?**	**Method used**	**Results**
Copy Number Variation	Yes	aCGH	All CNVs reported for each line
Whole Genome Sequencing (can be performed with GP2)	Yes	NGS	Available on request (Ryan Davis, USyd)
p53 function or sequence	Not analyzed	N/A	N/A
BCL-2 Status	Not analyzed	aCGH	N/A

PRKN 01-060 C9

Minimum iPSC Quality Control Panel			
**Genomic Baseline QC**	**Pass QC?**	**Method used**	**Results**
Genotyping	Yes	WGS	Exon deletion breakpoints and point mutation confirmed
Karyotype	Yes	High-denity SNP array (Illumina GSA24)	No significant CNV or BAF deviations detected, resolution ~25kbps
Pluripotent phenotype	Yes	PSC marker staining	Phase-contrast iPSC colony morphology, positivity for nuclear OCT4/POU5F1, NNAOG
Cell Line Identity	Yes	STR (10 markers)	Cell line genetic identity with source fibroblasts confirmed
Mycoplasma Testing	Yes	MycoAlert ELISA	Data not shown
Clonality	Yes	double-picked, +10 passage screened	Data not shown
Stability	Yes	30+ passage screened	Data not shown
**Extended iPSC Quality Control Panel**			
**Genomic Baseline QC**	**Pass QC?**	**Method used**	**Results**
Copy Number Variation	Yes	aCGH	All CNVs reported for each line
Whole Genome Sequencing (can be performed with GP2)	Yes	NGS	Available on request (Ryan Davis, USyd)
p53 function or sequence	Not analyzed	N/A	N/A
BCL-2 Status	Not analyzed	aCGH	N/A

PRKN 6 C4

Minimum iPSC Quality Control Panel			
**Genomic Baseline QC**	**Pass QC?**	**Method used**	**Results**
Genotyping	Yes	WGS, Allele-specific Sanger sequencing	Missense variant and exon deletion confirmed
Karyotype	Yes	High-denity SNP array (Illumina GSA24)	Normal
Pluripotent phenotype	Yes	PSC marker staining, differentiation	Pavan...Ovchinnikov, Stem Cell Res DOI: 10.1016/j.scr.2023.103211
Cell Line Identity	Yes	Allele Sanger haplotype sequencing	Cell line confirmed
Mycoplasma Testing	Yes	MycoAlert ELISA	Data not shown
Clonality	Yes	double-picked, +10 passage screened	Data not shown
Stability	Yes	10+ passage screened	Data not shown
**Extended iPSC Quality Control Panel**			
**Genomic Baseline QC**	**Pass QC?**	**Method used**	**Results**
Copy Number Variation	Yes	aCGH	All CNVs reported for each line.
Whole Genome Sequencing (can	Yes	NGS	Available on request (Ryan Davis, USyd)
p53 function or sequence	Not analyzed	N/A	N/A
BCL-2 Status	Not analyzed	aCGH	N/A

Table S2. QC testing of new *PRKN* lof iPSC. List of quality control tests performed on newly generated stem cell lines used in this publication. NA = not available

Antibody	Company	Catalogue no.	Species	Assay	Dilution factor
BRN2	Santa Cruz	Sc-6029	Goat	ICC	1:400
TBR1	Abcam	Ab31940	Rabbit	ICC	1:400
CTIP2	Abcam	Ab18465	Rat	ICC	1:400
MAP2	Thermofisher	PA1-10005	Chicken	ICC	1:500
Total α-syn	BD biosciences	610787	Mouse	ICC	1:400
				WB	1:1000
S129 phospho-α-syn	Abcam	Ab51253	Rabbit	ICC	1:300
				WB	1:1000
Total Tau (Tau5)	Novus Biologicals	NBP2-81091	Rabbit	ICC	1:300
Phospho-Tau (AT8)	Thermofisher	MN1020	Mouse	ICC	1:300
P62 / SQSTM1	Abcam	Ab56416	Mouse	ICC	1:400
				WB	1:1000
Galectin-3	Abcam	Ab2785	Mouse	ICC	1:400
TFEB	Abcam	Ab267351	Rabbit	ICC	1:400
LAMP2	Abcam	Ab25631	Mouse	ICC	1:400
FoxA2	Thermofisher	H00003170-M01	Mouse	ICC	1:400
TH	Thermofisher	PA1-5679	Sheep	ICC	1:500
LC3B	Abcam	Ab192890	Rabbit	ICC	1:400
β-actin	Abcam	Ab6726	Mouse	WB	1:10000
GBA	Enzo Life Sciences	H00002629-M01	Mouse	WB	1:1000
Total Rab-10	Cell Signaling Technology	8127S	Rabbit	WB	1:1000
PARKIN	Cell Signaling Technology	4211	Mouse	WB	1:1000

Table S3. Antibodies. Details for primary antibodies employed for immunocytochemistry and western blotting in this study.

Antibody	Species	Company	Catalogue no.	Application	Dilution factor
Anti-Mouse IgG (H + L) HRP Conjugate	Goat	Bio-Rad	1706516	WB	1:5000
Anti-Rabbit IgG (H + L) HRP Conjugate	Goat	Bio-Rad	1706515	WB	1:5000
Anti-Mouse IgG Highly Cross-adsorbed Alexa Fluor 647	Donkey	Thermofisher	A31571	ICC	1:500
Anti-Mouse IgG Highly Cross-adsorbed Alexa Fluor 488	Donkey	Thermofisher	A21202	ICC	1:500
Anti-Mouse IgG Highly Cross-adsorbed Alexa Fluor 568	Donkey	Thermofisher	A10037	ICC	1:500
Anti-Rabbit IgG Highly Cross-adsorbed Alexa Fluor 488	Donkey	Thermofisher	A21206	ICC	1:500
Anti-Rabbit IgG Highly Cross-adsorbed Alexa Fluor 568	Donkey	Thermofisher	A10042	ICC	1:500
Anti-Chicken IgY (H + L) Highly Cross-adsorbed	Donkey	Sigma-Aldrich	SAB4600031	ICC	1:500
Anti-Sheep IgG Cross Adsorbed Alexa Fluor 488	Donkey	Thermofisher	A11015	ICC	1:500
Anti-Rat IgG (H + L) Highly Adsorbed Alexa Fluor Plus 647	Donkey	Thermofisher	A48272	ICC	1:500
Anti-Goat IgG (H+L) Highly Cross-Adsorbed, Alexa Fluor Plus 647	Donkey	Thermofisher	A32849	ICC	1:500

Table S4. Secondary Antibodies. Details for secondary antibodies employed for immunocytochemistry and immunoblot in this study.

Stain / reagent	Company	Catalogue no.	Dilution
Cytopainter green (cytoplasmic stain)	Abcam	Ab176735	1:500
DQ red BSA	Thermofisher	D12051	1:100
PFB-FDGlu substrate	Thermofisher	P11947	1:1000

Table S5. Live cell imaging probes. Details for the live cell imaging probes employed in this study.

	Measurement / Experiment	Magnification / Objective	Number of wells per plate per cell line	Z-stack: Number of planes	Z-stack: Step size	Number of fields of view imaged per well	Number of cells imaged per well average (SEM)	Channel/antibody combinations
Live cell imaging	**GCase activity: PFB-FDGlu expression**	20x Water	6	3	1.5 µm	25	2382 (125)	Ch1: HOECHST Ch2: Alexa 488/PFB-FDGlu
	**Lysosomal activity: DQ-red BSA expression**	40x Water	3	3	0.5 µm	25	720 (73)	Ch1: HOECHST Ch2: Alexa 488/CytoGreen Ch3: Alexa 555/DQ-red-BSA
Fixed cells staining	**Characterization: TBR1 expression**	20x Water	2	7	1.5 µm	25	3898 (414)	Ch1: DAPI Ch2: Alexa 488/MAP2 Ch3: Alexa 555/TBR1
	**Characterization: BRN2 and CTIP2 expression**	20x Water	2	7	1.5 µm	25	3734 (373)	Ch1: DAPI Ch2: Alexa 488/CTIP2 Ch3: Alexa 555/BRN2 Ch4: Alexa 647/MAP2
	**ALP marker: P62**	40x Water	6	10	0.5 µm	49	1830 (145)	Ch1: DAPI Ch2: Alexa 488/MAP2 Ch3: Alexa 555/LC3B Ch4: Alexa 647/P62
	**Pathology: α-synuclein + phospho-α-synuclein expression**	40x Water	3	8	0.5 µm	49	2472 (197)	Ch1: DAPI Ch2: Alexa 488/MAP2 Ch3: Alexa 555/p-α-syn Ch4: Alexa 647/α-syn
	**Pathology: Tau 5 expression**	40x Water	3	8	0.5 µm	49	1726 (147)	Ch1: DAPI Ch2: Alexa 488/MAP2 Ch4: Alexa 647/Tau5
	**Pathology: phospho-Tau (AT8)**	40x Water	3	8	0.5 µm	49	1748 (163)	Ch1: DAPI Ch2: Alexa 488/MAP2 Ch4: Alexa 647/AT8
	**Lysosomal biogenesis and ruptured lysosomes: Galectin-3 and TFEB**	40x Water	3	10	0.5 µm	49	1475 (199)	Ch1: DAPI Ch2: Alexa 488/MAP2 Ch3: Alexa 555/TFEB Ch4: Alexa 647/Gal3

Table S6. Cortical neuron imaging parameters. Details for the acquisition of images from cortical neurons.

	Measurement / Experiment	Magnification / Objective	Number of wells per plate per cell line	Z-stack: Number of planes	Z-stack: Step size	Number of fields of view imaged per well	Number of cells imaged per well Average (SEM)	Channel/antibody combinations
**Live cell imaging**	**GCase activity: PFB-FDGlu expression**	20x Water	6	4	0.8 µm	8	1236 (268)	Ch1: HOECHST<br>Ch2: Alexa 488/PFB-FDGlu
	**Lysosomal activity: DQ-red BSA expression**	20x Water	3	4	0.8 µm	9	972 (151)	Ch1: HOECHST<br>Ch2: Alexa 488/CytoGreen Ch3: Alexa 555/DQ-red-BSA
**Fixed cells staining**	**Characterization: TH and FoxA2 expression**	20x Water	3	9	0.8 µm	8-14	1404 (250)	Ch1: DAPI<br>Ch2: Alexa 488/MAP2<br>Ch3: Alexa 555/TH<br>Ch4: Alexa 647/ FoxA2
	**ALP marker: P62**	40x Water	6	7	0.5 µm	8-14	1623 (250)	Ch1: DAPI<br>Ch2: Alexa 488/TH<br>Ch3: Alexa 555/P62<br>Ch4: Alexa 647/LC3B
	**Pathology: α-synuclein + phospho-α-synuclein expression**	40x Water	3	10	0.5 µm	8-14	1448 (189)	Ch1: DAPI<br>Ch2: Alexa 488/TH<br>Ch3: Alexa 555/α-syn<br>Ch4: Alexa 647/p-α-syn
	**Pathology: Tau 5 expression**	40x Water	3	10	0.5 µm	8-14	2330 (430)	Ch1: DAPI<br>Ch2: Alexa 488/TH<br>Ch4: Alexa 555/Tau5
	**Pathology: phospho-Tau (AT8)**	40x Water	3	10	0.5 µm	8-14	928 (102)	Ch1: DAPI<br>Ch2: Alexa 488/TH<br>Ch4: Alexa 647/AT8
	**Lysosomal biogenesis and CMA: Galectin-3 and LAMP2A**	40x Water	3	10	0.5 µm	8-14	1312 (230)	Ch1: DAPI<br>Ch2: Alexa 488/TH<br>Ch3: Alexa 555/Gal3<br>Ch4: Alexa 647/LAMP2

Table S7. Ventral midbrain neuron imaging parameters. Details for the acquisition of images from ventral midbrain neuron cultures.
