# Supplementary figures and images for: Dopamine and cortical neurons with different Parkinsonian mutations show variation in lysosomal and mitochondrial dysfunction
Source: NPJ Parkinsons Dis. 2025 Jun 20;11:177. doi: 10.1038/s41531-025-01048-2 (PMC12181412; doi:10.1038/s41531-025-01048-2)

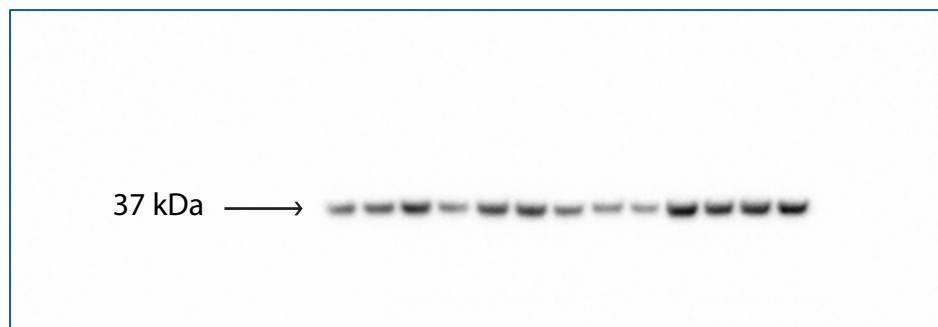

$\beta$ -actin

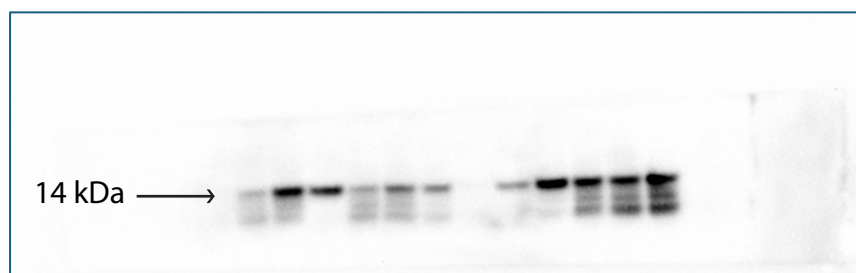

$\alpha$ -syn

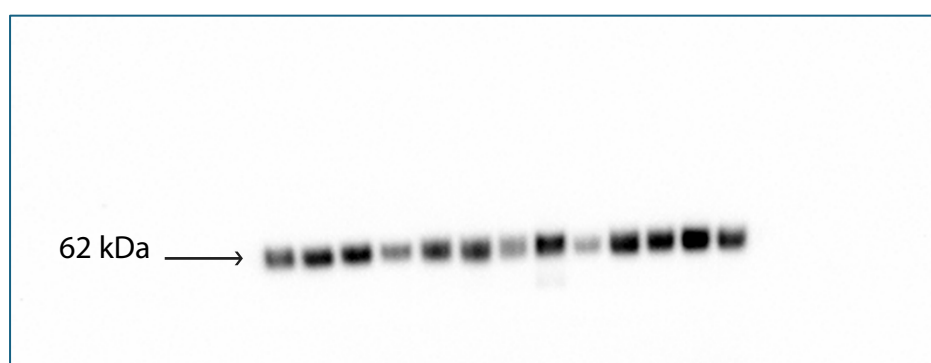

GBA

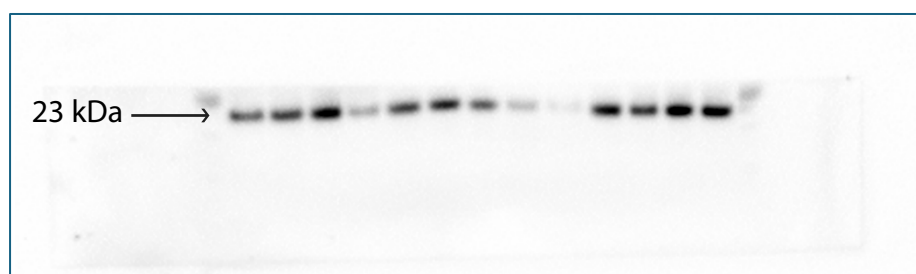

Total Rab -10

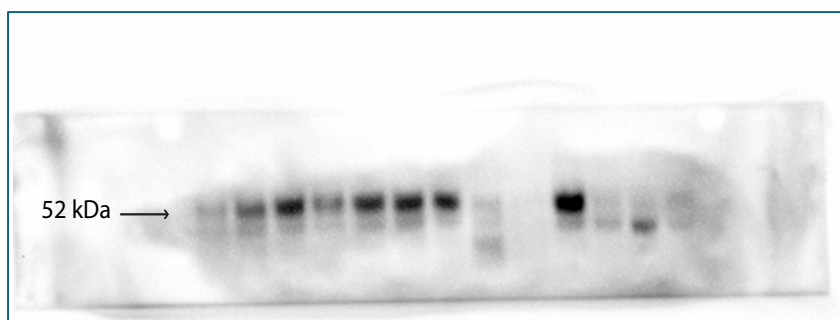

PARKIN

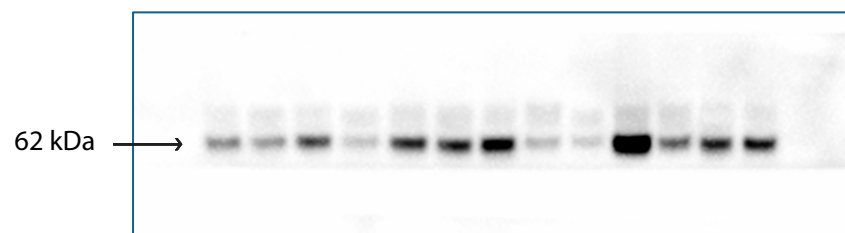

P62

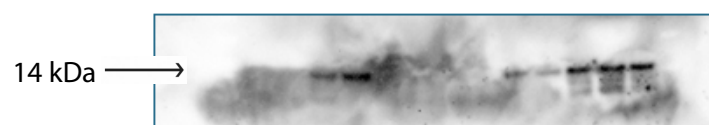

S129 p- $\alpha$ -syn

Supplement: Supplementary file 2 — uncropped WB [file 41531_2025_1048_MOESM2_ESM.pdf]
